# Mutation profiling of tumor DNA from plasma and tumor tissue of colorectal cancer patients with a novel, high-sensitivity multiplexed mutation detection platform

**DOI:** 10.18632/oncotarget.3041

**Published:** 2014-12-10

**Authors:** Evelyn Kidess, Kyra Heirich, Matthew Wiggin, Valentina Vysotskaia, Brendan C. Visser, Andre Marziali, Bertram Wiedenmann, Jeffrey A. Norton, Mark Lee, Stefanie S. Jeffrey, George A. Poultsides

**Affiliations:** ^1^ Department of Surgery, Stanford University School of Medicine, Stanford, CA, USA; ^2^ Boreal Genomics, Mountain View, CA, USA and Vancouver, BC, Canada; ^3^ Deparment of Medicine, Division of Hepatology and Gastroenterology, Charité University Hospital, Berlin, Germany

**Keywords:** biomarker, circulating tumor DNA, colon cancer, ctDNA, hepatic metastasis

## Abstract

**BACKROUND:**

Circulating tumor DNA (ctDNA) holds promise as a non-invasive means for tumor monitoring in solid malignancies. Assays with high sensitivity and multiplexed analysis of mutations are needed to enable broad application.

**METHODS:**

We developed a new assay based on sequence-specific synchronous coefficient of drag alteration (SCODA) technology, which enriches for mutant DNA to achieve high sensitivity and specificity. This assay was applied to plasma and tumor tissue from non-metastatic and metastatic colorectal cancer (CRC) patients, including patients undergoing surgical resection for CRC liver metastases.

**RESULTS:**

Across multiple characterization experiments, the assay demonstrated a limit of detection of 0.001% (1 molecule in 100,000) for the majority of the 46 mutations in the panel. In CRC patient samples (n=38), detected mutations were concordant in tissue and plasma for 93% of metastatic patients versus 54% of non-metastatic patients. For three patients, ctDNA identified additional mutations not detected in tumor tissue. In patients undergoing liver metastatectomy, ctDNA anticipated tumor recurrence earlier than carcinoembryonic antigen (CEA) value or imaging.

**CONCLUSIONS:**

The multiplexed SCODA mutation enrichment and detection method can be applied to mutation profiling and quantitation of ctDNA, and is likely to have particular utility in the metastatic setting, including patients undergoing metastatectomy.

## INTRODUCTION

Although the existence of circulating tumor DNA (ctDNA) was first reported more than 30 years ago [1], interest in the practical application of ctDNA has only recently accelerated with the development of technologies suited to detecting and measuring this analyte [2-4]. Tumor monitoring with ctDNA may offer a non-invasive approach to assess microscopic residual disease, response to therapy, and tumor molecular profiles in the context of intratumoral heterogeneity and clonal evolution [5, 6].

For colorectal cancer (CRC) patients, there have been recent advances in screening, surgical techniques, and use of chemotherapeutic and biologic agents, which have significantly prolonged survival [7-9]. A deeper understanding of this disease, particularly the importance of *KRAS* mutational status in conferring resistance to therapies directed against the epidermal growth factor receptor (EGFR), has also enabled molecularly-guided treatment strategies [9]. Still, improved methods for monitoring disease burden and tumor molecular profiles of CRC are needed to optimize detection strategies and use of existing therapies, as well as to accelerate development of new treatments. Conventional monitoring of CRC is primarily based on cross-sectional imaging and measurement of serum carcinoembryonic antigen (CEA). Both of these methods can be associated with false positives: inflammatory conditions such as diverticulitis or inflammatory bowel disease can lead to elevated levels of CEA [10-12], while benign conditions can mimic malignant lesions on imaging and thereby necessitate confirmatory biopsy [13, 14]. Imaging and CEA are also associated with false negatives, since subcentimeter lesions (e.g. at the periphery of ablated liver metastases) may not be detected by imaging [13, 14], and a subset of patients with advanced stage CRC may not show elevated levels of CEA [11]. For both methods, a significant further limitation is that neither provides information about the molecular profile of the disease. Improved tumor monitoring tools may be particularly important for patients with resectable metastatic disease, where a subset of patients can achieve long-term disease-free survival [15, 16]. Better assessment of residual disease and evolving changes in tumor molecular profiles may enable improved risk stratification and tailoring of perioperative therapy in metastatic CRC.

Multiple methods have been developed to enable the assessment of ctDNA in CRC, including digital PCR, ‘BEAMing’ (beads, emulsion, amplification, and magnetics) and other approaches based on PCR and next-generation sequencing [2-4]. Several studies have shown that for patients with identifiable *KRAS* mutations in their tumor tissue, the corresponding mutations can be detected in DNA isolated from plasma, and elevated ctDNA levels have been associated with decreased overall 2-year survival [4, 17, 18]. Furthermore, acquired resistance to EGFR-inhibitors due to emergence of mutations in *KRAS*, *NRAS*, and other genes can be monitored non-invasively by serial assessment of ctDNA [19, 20]; resistance mutations can be identified in plasma DNA up to ten months earlier than conventional imaging reveals recurrence [19].

To date, most studies have analyzed plasma samples from patients with advanced stage CRC, as the abundance of ctDNA has been found to increase with disease progression; ctDNA has not been reliably detected in a significant proportion of patients with early stage disease [3, 17, 18, 21]. For clinical applications such as assessment of residual disease, surveillance, and early detection, specialized techniques with high sensitivity and specificity will thus need to be developed for the detection and quantification of mutant alleles in these settings [2, 3]. The ability to reliably detect ctDNA in CRC patients with minimal residual disease would enhance the clinical utility of ctDNA, not only for CRC tumor monitoring, but also for therapeutic decision-making.

To address this need, we have developed a new assay for the detection of ctDNA across a panel of cancer-related mutations with high sensitivity and specificity based upon sequence-specific synchronous coefficient of drag alteration (SCODA) technology [22], which enables efficient enrichment of mutant DNA from plasma. To evaluate this new assay, we analyzed tissue and plasma samples from a cohort of patients with non-metastatic and metastatic CRC. Additionally, we performed an exploratory analysis of longitudinal ctDNA measurements in patients undergoing surgical resection for hepatic metastatic disease.

## RESULTS

### Development and characterization of multiplexed SCODA mutation enrichment and detection platform

**Figure 1:**
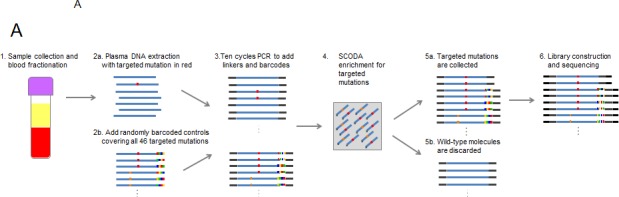

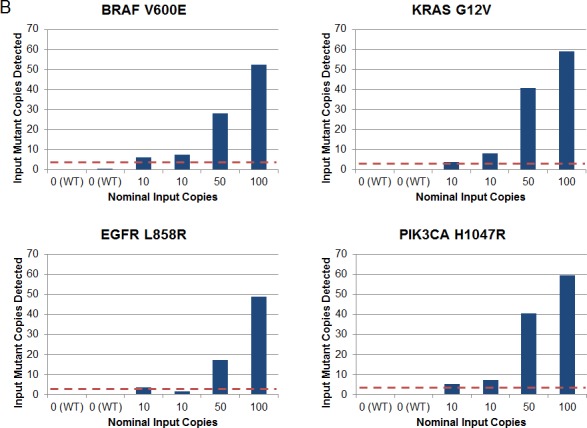
Multiplexed SCODA mutation enrichment and detection assay A) Method. Following plasma preparation (1), plasma DNA is extracted (2a) and spiked with randomly barcoded internal controls (2b) representing every mutation in the panel, and amplified with a limited number of cycles in a multiplex PCR reaction (3). PCR primers are designed to amplify regions of the genome containing the 46 mutations of interest, and carry universal linker sequences and sample barcodes to enable subsequent sample processing and multiplexing. Amplified DNA and internal controls are enriched for the 46 mutations of interest by SCODA cyclic electrophoresis (4) in a gel containing hybridization probes against all mutations of interest. DNA enriched for mutant sequences (5a) is amplified through additional PCR as per conventional Illumina library construction, pooled with additional samples, and sequenced (6). B) Assay characterization. Each graph denotes results from samples created by titrating synthetic DNA carrying the specified mutation into reference wild-type (WT) DNA. Horizontal axes denote estimated input copies per sample, while the vertical axes denote the number of copies detected by the assay. Limit of Detection (LOD) is calculated as the greater of a single copy of mutant DNA or three standard deviations above the average background detected for each mutation in wild-type DNA samples. Input copies are limited to 10 and greater to limit the effect of sampling fluctuation in the titration. Each sample is 300 ng of DNA, such that 10 copies are equivalent to an allele fraction of ~ 0.01%.

To develop an assay with high sensitivity and specificity across a large panel of defined mutations, we engineered a multiplexed version of the previously described sequence-specific synchronous coefficient of drag alteration (SCODA) assay (Figure 1A) [22]. This methodology overcomes issues of low specificity introduced by error rates in conventional PCR and sequencing techniques by simultaneously enriching for multiple mutant DNA sequences based on repetitive transient hybridization (see Supplementary Material).

Initial implementation of this multiplexed SCODA mutation enrichment and detection assay focused on 46 mutations in 4 genes (*BRAF*, *EGFR*, *KRAS*, and *PIK3CA*), constituting a set of commonly encountered mutations of clinical relevance in colorectal and non-small cell lung cancers (Table 1) [15, 23-31]. For each individual mutation, the assay underwent a series of analytical characterization experiments for specificity and sensitivity performed on reference wild-type DNA samples supplemented with defined quantities of synthetic DNA molecules carrying the corresponding mutant sequences. Analysis of control samples (without synthetic mutant DNA) demonstrated very low background signal, below the single molecule limit for the vast majority of assessments (Supplementary Material). In experiments with defined inputs of mutant sequences, the assay was able to consistently detect 10 or fewer input molecules of mutant DNA (Figure 1B). These characterization experiments enabled definition of the limit of detection (LOD) for each of the 46 mutations in the assay, which, for most mutations, represents as little as one copy of mutant DNA in plasma samples (Table 1 and Supplementary Material). Assessment of the assay in a set of healthy volunteers without a history of cancer revealed no detectable mutant DNA sequences in 43 of 47 subjects, with the remaining 4 cases showing signal at or near the LOD (Supplementary Material).

**Table 1:** Genes, mutations, and Limit of Detection (LOD) of multiplexed SCODA mutation detection assay

Mutation	LOD
BRAF V600D	0.001%
BRAF V600E (1799T>A)	0.002%
BRAF V600E (1799_1800TG>AA)	0.001%
BRAF V600K	0.001%
EGFR E746_A750del_2235	0.001%
EGFR E746_A750del_2236	0.001%
EGFR E746_A750>IP	0.001%
EGFR E746_P753>VS	0.001%
EGFR E746_S752>A	0.001%
EGFR E746_S752>D	0.001%
EGFR E746_S752>I	0.001%
EGFR E746_S752>V	0.001%
EGFR E746_T751>A	0.001%
EGFR E746_T751>I	0.001%
EGFR E746_T751>IP	0.001%
EGFR E746_T751>V	0.001%
EGFR E746_T751>VA	0.001%
EGFR E746_T751del	0.001%
EGFR K745_E749del	0.001%
EGFR L747_A750>P_2238	0.001%
EGFR L747_A750>P_2239	0.001%
EGFR L747_E749del	0.001%
EGFR L747_P753>Q	0.001%
EGFR L747_P753>S	0.001%
EGFR L747_S752>Q	0.001%
EGFR L747_S752del	0.001%
EGFR L747_T751>P	0.001%
EGFR L747_T751>Q	0.001%
EGFR L747_T751>S	0.001%
EGFR L747_T751del	0.001%
EGFR T790M	0.020%
EGFR L858R (2573T>G)	0.001%
EGFR L858R (2573_2574TG>GT)	0.001%
KRAS G12A	0.003%
KRAS G12C	0.001%
KRAS G12D	0.020%
KRAS G12R	0.001%
KRAS G12S	0.018%
KRAS G12V	0.001%
KRAS G13C	0.008%
KRAS G13D	0.018%
PIK3CA E542K	0.023%
PIK3CA E545K	0.024%
PIK3CA Q546K	0.007%
PIK3CA H1047L	0.001%
PIK3CA H1047R	0.002%

The table shows the specificity limit of detection (LOD) for the assay, i.e. the minimum mutational abundance that can be confidently detected. The specificity limit is determined by performing the assay over multiple runs on multiple lots of reference wild-type DNA samples to calculate the average and standard deviation of the wild-type signal. For each mutation, LOD is defined as this average background signal + 3 standard deviations; in cases where the calculated LOD would be less than 1 molecule, the LOD is set to 1 molecule. For example, for 300 ng of total input DNA, a LOD of 0.001% corresponds to 1 mutant molecule in 100,000 genome equivalents

### Detection of plasma ctDNA in non-metastatic and metastatic colorectal cancer patients

As an initial application of this new mutation detection platform, we performed an exploratory analysis of tumor and pre-operative plasma samples from 38 CRC patients undergoing surgery (Table 2). This cohort included patients with non-metastatic (stage I-III, n=19) and metastatic (stage IV, n=19) disease. Median age was 63 years and 61% of patients were male. Among the stage IV patients, metastatic disease was most commonly confined to the liver (16/19). Over half (10/19) of the metastatic patients received preoperative systemic therapy, whereas none of the patients with non-metastatic disease received pre-operative treatment.

**Table 2:** Demographics and clinical characteristics of study patients

Characteristic	No. (%) (total n=38)
Age	39 – 89 yrs (Median 63)
Gender	
Male	23 (61%)
Female	15 (39%)
Stage	
I	5 (13%)
II	11 (29%)
III	3 (8%)
IV	19 (50%)
For stage IV patients (n=19)	
Pattern of metastatic spread	
Liver only	16
Liver and peritoneal	2
Ovary	1
Preoperative chemotherapy	
None	9
1 regimens	8
2+ regimens	2

**Figure 2:**
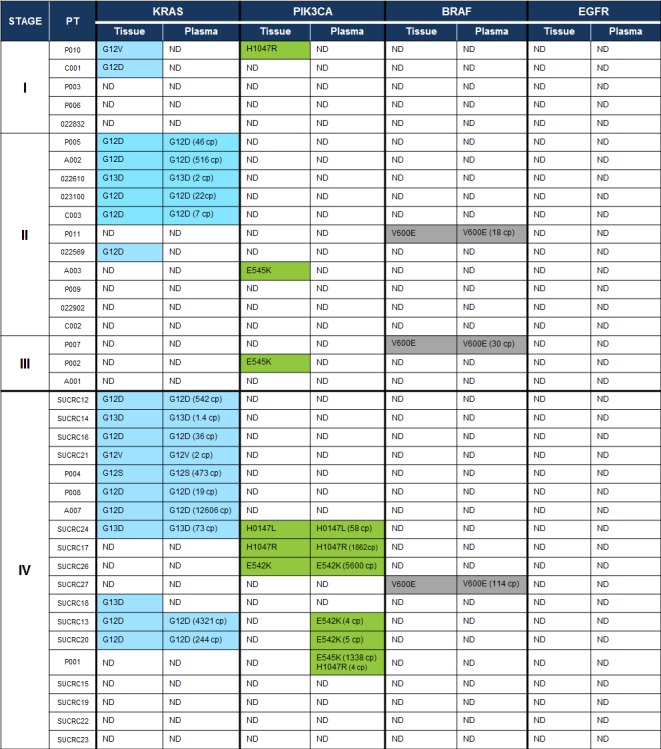
Mutations detected in tissue and plasma with the multiplexed SCODA mutation enrichment and detection assay In highlighted cells, the detected mutation allele (or alleles) is specified, and for plasma, the number of detected copies (cp) of mutant DNA is also provided, normalized for a 5 mL input volume of plasma. ND, not detected.

The multiplexed SCODA mutation detection assay was used to analyze extracted DNA from patient tumor tissue and pre-operative plasma samples for the presence of mutations in *KRAS*, *PIK3CA*, *BRAF* and *EGFR* as defined in our panel (Table 1). In tumor tissue, 68% of the cohort (26 of 38 patients) showed at least one detectable mutation from the panel, including 14 of 19 (74%) patients with metastatic disease and 12 of 19 (63%) patients with non-metastatic disease. The distribution of observed mutations was consistent with prior reports (Figure 2): 50% (19 of 38 patients) had a *KRAS* mutant tumor, 16% (6 of 38 patients) had a *PIK3CA* mutation, 8% (3 of 38 patients) showed a mutation in *BRAF*, and none showed an *EGFR* mutation. Of note, two patients harbored concurrent mutations in *KRAS* and *PIK3CA*. Importantly, the SCODA mutation detection platform demonstrated excellent concordance (18/19 cases tested, 95%) with a conventional quantitative PCR assay for *KRAS* performed on tumor tissue, a standard-of-care assessment for patients with metastatic CRC (Table 3). No discordances were observed when the conventional assay identified a *KRAS* mutation and the SCODA assay did not; the only discordance was a case where the SCODA assay found a very low *KRAS* mutant signal in tissue, at a level below the reported sensitivity for conventional *KRAS* PCR assays [32].

**Table 3:** KRAS mutational status of tumor tissue from metastatic colorectal cancer patients determined by multiplexed SCODA mutation detection assay vs. conventional quantitative PCR method

Patient	KRAS Mutation Status	
	Multiplexed SCODA mutation detection	Conventional quantitative PCR
SUCRC12	G12D (41%)	G12D
SUCRC14	G13D (0.04%)	WT
SUCRC16	G12D (6.5%)	G12D
SUCRC21	G12V (4.75%)	G12V
P004	G12S (24%)	G12S
P008	G12D (17%)	G12D
A007	G12D (36%)	G12D
SUCRC24	G13D (1.7%)	G13D
SUCRC17	WT	WT
SUCRC26	WT	WT
SUCRC27	WT	WT
SUCRC18	G13D (3.8%)	G13D
SUCRC13	G12D (18%)	G12D
SUCRC20	G12D (0.285%)	G12D
P001	WT	WT
SUCRC15	WT	WT
SUCRC19	WT	WT
SUCRC22	WT	Not analyzed
SUCRC23	WT	WT

Note: WT, wild-type.

By comparison, the multiplexed SCODA mutation detection assay detected mutant DNA in the plasma of 53% (21 of 38) of CRC patients, including 20 of 26 patients (77%) found to have mutations in tumor tissue (Figure 2), with the identical allele detected in both tissue and plasma. Tissue/plasma concordance differed markedly between the metastatic and non-metastatic cases. In metastatic cases (stage IV), 14 of the 15 mutations (93%) identified in tumor tissue (14 patients) were also found in plasma. In contrast, for the non-metastatic patients (stages I-III), only 7 of the 13 mutations (54%) identified in tumor tissue samples (12 patients) could be detected in plasma. For the stage I patients, none of the three mutations observed in tissue were detectable in the corresponding plasma samples. Interestingly, in three of the patients with metastatic disease, a mutation was detected in plasma that was not observed in tissue; in all of those cases, the plasma-specific mutations were found in the *PIK3CA* gene.

Differences in the detection of ctDNA in metastatic and non-metastatic patients were not influenced by the amount of total cell-free DNA in the plasma, as levels of recovered cell-free DNA were not statistically different between these groups (p=0.096 by t-test), or between patients with and without detectable ctDNA (p=0.26 by t-test) (data not shown).

### Perioperative monitoring of ctDNA in stage IV colorectal cancer patients with potentially resectable disease

For the subset of patients with potentially resectable metastatic disease and detectable mutations by the SCODA assay in pre-operative plasma, we further analyzed post-operative plasma samples obtained on the fifth post-operative day and at routine follow-up visits in the context of CEA levels, cross-sectional imaging, and post-operative therapy. In particular, we sought to explore how ctDNA levels might be useful for this specific population of patients, potentially to inform the completeness of surgical resection and response to subsequent therapy, as well as to anticipate the recurrence of disease.

**Figure 3:**
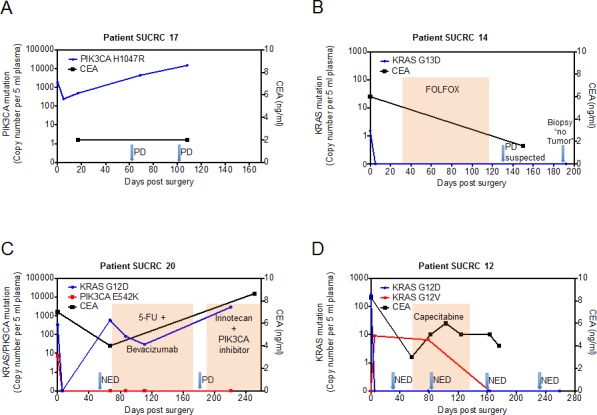
Perioperative dynamics of plasma mutation levels in patients with stage IV colorectal cancer Panels A-D show plasma levels of detected mutations in the circulating tumor DNA (ctDNA, left y-axis) as well as CEA values (right y-axis) in patients that underwent surgery with the intent of complete metastasis resection. The value at time point 0 represents the preoperative ctDNA level. ctDNA levels (cp, copies) are normalized for 5 mL input volume of plasma. After blood draw, surgery was performed at day 0. Arrows indicate imaging assessments by computed tomography (CT). In panels B, C and D, colored shading indicates the administration of chemotherapy. PD, progressive disease; NED, no evidence of disease. CEA normal range is 0-5 ng/mL.

Figure 3 shows this longitudinal assessment of disease course for four illustrative patients undergoing surgical resection of metastatic disease. All four patients had detectable levels of ctDNA in pre-operative plasma, which declined following surgery, suggesting that ctDNA levels are generally reflective of disease burden. For patient SUCRC17 (Figure 3A), the plasma level of *PIK3CA* H1047R mutant DNA fell nearly 10-fold following surgery but remained detectable at high levels; this patient developed rapid disease recurrence on imaging, with concomitant rapidly rising ctDNA levels, despite a constantly normal CEA level. By contrast, for the other three patients (SUCRC14, SUCRC20, and SUCRC12; Figures 3B, 3C, and 3D, respectively), mutations detected in pre-operative plasma DNA were not detectable at the first assessment following surgery, presumably reflecting complete removal of metastatic lesions. The follow-up course for these three patients, however, revealed important differences in the relationships of ctDNA levels with clinical findings. Patient SUCRC14 had no detectable ctDNA in follow-up plasma at day 192, confirming a normal CEA value on an earlier follow-up, and demonstrated no evidence of disease recurrence; suspected progression of disease on imaging at day 132 was deemed a false positive after a biopsy showed no evidence of tumor (Figure 3B). Similarly, for patient SUCRC20, the two detectable mutations (*KRAS* G12D and *PIK3CA* E542K) in pre-operative plasma were not detected in the immediate post-operative period; however, follow-up assessments identified re-emergence of the *KRAS* G12D mutant DNA in plasma two months after surgery, while imaging and CEA levels suggested no evidence of recurrence. The patient ultimately developed progressive disease on imaging, as well as rising levels of CEA and ctDNA, despite the administration of systemic therapy (Figure 3C). Patient SUCRC12 had a detectable *KRAS* mutation in pre-operative plasma (G12D), which became undetectable following surgery (Figure 3D); however, a distinct *KRAS* mutation (G12V) became evident in plasma samples taken following surgery and also during follow up, 80 days later. No definitive imaging or CEA level correlate was identified for this signal, and following a course of capecitabine, the *KRAS* G12V mutant DNA was no longer detectable in plasma.

For the patients with two mutations found in ctDNA (SUCRC20 and SUCRC12), the dynamics of the mutations seen during follow-up may be indicative of clonal heterogeneity and evolution. For patient SUCRC20 in particular, the preoperatively analyzed plasma sample had concurrent mutations in *KRAS* and *PIK3CA*, but plasma samples taken during disease progression carried only the *KRAS* mutation; the absence of the *PIK3CA* mutation at these later timepoints may have implications for the selection of therapy, including the likelihood of response to targeted agents. It is also notable that, for patients SUCRC17, SUCRC20 and SUCRC12, elevated ctDNA levels appeared to anticipate disease progression better and at earlier time points than CEA levels. Although confirmation in additional large studies will be required, these observations suggest potential applications for ctDNA in the management of patients following resection of metastatic disease, with both prognostic and tumor monitoring utility, which should at least complement existing clinical tools.

## DISCUSSION

In this study, we introduced a novel multiplexed SCODA mutation detection platform that identifies ctDNA in patient plasma with high sensitivity and specificity. Application of this assay in an exploratory analysis of CRC patients revealed lower rates of ctDNA detection in plasma from non-metastatic patients, despite the analytical capabilities of our assay, suggesting a biological cause. By contrast, the assay reliably detected ctDNA in the plasma of patients with metastatic disease, and longitudinal assessments in metastatic patients undergoing surgical resection and chemotherapy provide a rationale for prognostic and tumor monitoring applications, which warrant further study in this patient population.

In some clinical settings, the measurement of plasma ctDNA can be very challenging due to the low level of signal and the high background level of circulating wild-type DNA. Through enrichment of mutant alleles, the SCODA method effectively removes background wild-type DNA, leading to a reduction in the false-positive rate and ultimately enabling detection of as little as single copies of mutant DNA. As such, this method is extremely sensitive (with a limit of detection as low as 0.001%) that meets – and in some cases exceeds – what has previously been reported for droplet digital PCR, BEAMing, and next generation sequencing approaches [3, 4, 33, 34]. Importantly, this level of sensitivity and specificity is achieved without previous knowledge of each patient's tumor mutation profile from tissue, which, together with multiplexed assessment of a large and expandable panel of defined mutations, should readily enable generalized application without patient-specific assay development, as is required for other methods [3, 35].

Several groups have studied ctDNA in non-metastatic and metastatic CRC patients [3, 4, 17-19, 36]. Our observed concordance of 54% between tissue and plasma mutations in non-metastatic patients is consistent with previous reports [17, 18, 36]. Bettegowda *et al.* used a combination of methods, including BEAMing and SafeSeq [34, 37], to show that 49-78% of patients with localized disease had detectable ctDNA [17]. Lecomte *et al.* found that 63% of a group of stage I, II and III CRC patients had ctDNA detectable by mutant allele-specific amplification directed against *KRAS* and methylation-specific PCR for *p16* [18]. Based on the technical performance of our assay, the absence of detectable ctDNA signal in the plasma of the early-stage patients appears to be likely related to differences in tumor burden, anatomy, or biology, rather than inadequate analytical sensitivity. Similar considerations have been described for circulating tumor cells, which are also much less abundant in early stage disease [38-42]. Associations of ctDNA levels with tumor burden, as evidenced by tumor stage and CEA for example, have been reported [3, 20], although ctDNA levels vary considerably within stage [17], suggesting additional contributory factors. In this regard, anatomic differences may also be explanatory, as early stage tumors may have less access to larger blood vessels owing to more limited depth of invasion of the bowel wall. Additionally, variation in tumor biology is also likely to underlie these differences, and may include properties of metastatic disease related to higher proliferative and apoptotic rate, and also lower efficiency of host mechanisms for clearing necrotic tumor cells [2, 43]. It is possible that higher sensitivity in early-stage disease could be achieved with a broader assessment of tumor molecular profiles, including early mutational events (e.g., *APC* and *TP53* which may be shared across all subclones) as well as copy-number variations and patterns of hyper- and hypomethylation [3, 18, 44, 45]. Nevertheless, the available data suggests that other methods for assaying ctDNA (such as by broad epigenetic panels) will need to be clinically tested to robustly address clinical questions for patients with early-stage disease.

In our metastatic CRC patients, we found a high concordance rate (93%) between plasma and tumor mutations, which is consistent with previous reports [4, 17]. These data suggest that ctDNA may be a meaningful non-invasive surrogate for tissue biopsies for patients with metastatic disease. In addition, in three cases we detected a *PIK3CA* mutation in plasma DNA which was not present in the corresponding metastatic tissue sample, indicating that ctDNA may further provide insight into tumor heterogeneity and thus avoid tumor sampling bias [46]. During the longitudinal assessment of ctDNA levels in patients with resectable liver metastases from CRC, we observed two cases where two different mutations were detected in plasma DNA, presumably reflecting different metastatic subclones, of which one re-emerged following surgery. As such, ctDNA may offer valuable real-time insight into the clonal tumor evolution during the course of treatment, and thus offer the opportunity to direct therapy to the dominant subclone at any given point in a patient's treatment course. Finally, our results additionally suggest that ctDNA levels following surgery for metastatic disease may be prognostic for disease recurrence. For patients with resectable metastatic disease, assessment of residual disease following surgery, along with the ability to anticipate recurrence and to gain insight into the molecular profile of the tumor tissue, may eventually enable optimization of post-operative therapy. All of these concepts warrant further study and will require further testing in larger patient cohorts.

Interpretation of these results should be caveated by the limitations of our study. Broader application of this technology in clinical practice will also require a larger panel of mutations with representation of additional genes relevant to CRC. In this regard, an expanded assay is currently under development. Furthermore, the patient cohort included in this exploratory study is relatively small, and therefore does not permit definitive conclusions. Nevertheless, this study represents proof of concept for the multiplexed SCODA assay for analyzing tumor mutations in plasma and is informative with respect to the technical challenges facing ctDNA assessment, particularly in early-stage disease, as well as for the detection of residual disease following surgical resection.

In this study, we present a multiplexed SCODA mutation detection assay for measuring plasma ctDNA, which has been characterized here for its ability to assess a panel of 46 mutations with very high sensitivity and specificity. Using this assay we provide evidence for ctDNA as a very promising non-invasive tool for real-time tumor molecular profiling in CRC, including the setting of resectable metastatic disease, where more accurate information about residual disease, prognosis and tumor molecular evolution should enable optimization of perioperative therapy. While our findings require further investigation, the technology platform we have described should enable studies designed to establish the utility of ctDNA in improving patient care across a variety of clinical settings.

## MATERIALS & METHODS

### Patients and sample collection

From September 2013 to September 2014, clinical data, tissue and plasma specimens were collected from a total of 38 patients with stage I, II, III, and IV colorectal cancer at multiple sites in the United States and Europe. Eligibility included histologically confirmed adenocarcinoma of the colon or rectum, availability of a plasma specimen drawn prior to surgery, and sufficient frozen tissue for molecular analysis. With the exception of 4 patients with advanced disease, all patients underwent surgery with curative intent, including 16 patients with resectable metastatic disease recruited at Stanford University Hospital. Additionally, plasma samples from 47 healthy donors were also evaluated. Blood samples were collected in Cell-Free DNA BCT tubes (Streck Inc., Omaha, NE) [47] pre-operatively, within 1-3 days prior to surgery. For a subset of patients undergoing curative resection of metastatic disease, additional blood collections were performed post-operatively and at follow-up visits. Tumor tissue was collected during surgery, transported on ice and stored at −80°C until further processing.

The study was conducted according to the ethics and Institutional Review Board standards of the participating hospitals and clinics. All patients provided written informed consent prior to being included into the study.

### Mutation enrichment and detection

The methodology for mutation enrichment and detection used in this study is based on the sequence-specific synchronous coefficient of drag alteration (SCODA) technology [22]. This multiplexed SCODA mutation enrichment and detection platform is depicted schematically in Figure 1. Full details of the mutation enrichment and detection workflow, including data analysis, are provided in the Supplementary Material.

Briefly, plasma was prepared by conventional protocols from 5-10 mL of whole blood from each patient, from which DNA was extracted. Synthetic internal positive controls (IPCs) for each mutation, used to calculate the process yield, to enable quantitation of mutant DNA, and to monitor assay performance, were then added to each sample. The resulting DNA sample was then subjected to a multiplex PCR reaction (at low amplification to minimize PCR errors prior to enrichment) with primers carrying sample-specific barcodes (to enable sample multiplexing) as well as universal linker sequences which serve as priming sites during library construction.

The amplified sample was then enriched for mutant DNA by a multiplexed SCODA mutation enrichment method on a device specifically engineered for this application. Following enrichment, a sequencing library was constructed by PCR using primers complementary to the universal linkers, and tagged with adaptors (Illumina, San Diego, CA). The library for each sample was then quantified by quantitative PCR, samples were pooled into sets of six, each having a unique sample barcode, and sequenced on the MiSeq platform (Illumina, San Diego, CA).

Forward and reverse reads from the MiSeq FastQ files were merged to give a single read for each cluster, de-multiplexed according to sample barcode and then aligned to the amplicons within the panel. Clusters were assigned mutant status if they contained one of the 46 mutations in the multiplexed SCODA mutation detection assay. Clusters arising from genomic DNA and IPC molecules were distinguished by the presence of random identifier sequences in the IPCs. IPC molecules were then binned according to the random identifier sequences, and the number of clusters arising from each unique combination of identifiers (random identifier sequences, barcodes, mutations) was counted. From these distributions, we measured the average cluster yield for each mutation in each sample, which was used to calculate the number of input mutant copies from the number of mutant clusters observed. To make mutation calls, the number of input mutant copies was compared to the limit of detection for each mutation, defined as 3 standard deviations above the mean background signal (observed over multiple runs of reference wild-type samples). Positive mutant calls were made only where at least a single mutant copy was observed and the abundance of the mutation (percentage with respect to all input genomes) was greater than the limit of detection.

## SUPPLEMENTARY MATERIAL


