# Supplementary material for: Mutation profiling of tumor DNA from plasma and tumor tissue of colorectal cancer patients with a novel, high-sensitivity multiplexed mutation detection platform
Source: Oncotarget. 2014 Dec 10;6(4):2549–61. doi: 10.18632/oncotarget.3041 (PMC4385870; doi:10.18632/oncotarget.3041)
Supplement: Supplementary file 1 [file oncotarget-06-2549-s001.pdf]

Mutation profiling of tumor DNA from plasma and tumor tissue of colorectal cancer patients with a novel, high-sensitivity multiplexed mutation detection platform

Supplementary Material

Laboratory analyses were performed at Boreal Genomics (Mountain View, CA and Vancouver, Canada). Genes and mutations included in the multiplexed SCODA assay described here were selected through a review of published literature for prevalent mutations in colorectal and lung cancer with clinical relevance [1-10]. In total, 46 mutations across 4 genes (*BRAF*, *EGFR*, *KRAS*, and *PIK3CA*) were included (Table 1).

DNA extraction

DNA was purified from tissue samples using the QIAmp DNA Mini Kit and from plasma using the QIAmp Circulating Nucleic Acid Kit (Qiagen N.V., Netherlands), according to the manufacturer's instructions. DNA was eluted in 180 µl buffer AVE and stored at -80°C.

Following DNA purification, the number of genome copies present in each DNA sample was assessed using quantitative PCR of 2 reference genomic loci (*COG5* and *ALB*), using two serial, 10-fold dilutions and performed in duplicate.

Mutation enrichment and sequencing workflow

Internal positive controls (IPCs), used to calculate the process yield and to monitor assay performance for each mutation individually, were then added to each sample. The IPCs have identical sequence to the mutant alleles at PCR primer and SCODA probe sites, but additionally contain random identifier sequences (RIDs), which are random DNA barcodes that facilitate yield calculations for individual input molecules and allow controls to be easily distinguished from mutant DNA sequences arising from plasma genomic DNA. Approximately 50 internal positive control molecules were added for each mutation in the 46-mutation panel. A negative control sample, containing 300 ng wild-type DNA (Roche Diagnostics, Indianapolis, IN), was run in parallel to the test samples.

Each sample was then assigned a unique sample DNA barcode in a multiplex 10-cycle barcoding PCR reaction with Q5 DNA polymerase (New England BioLabs, Ipswich, MA). 99% of each sample (up to a maximum of 300 ng DNA) was used as template to amplify 7 loci containing the 46 mutations: *BRAF* exon 15, *KRAS* exon 2, *PIK3CA* exons 9 and 20, and *EGFR* exons 19, 20, and 21. The remaining 1% of the sample DNA was barcoded in a separate reaction in which mutation panel loci and two additional control loci (*COG5* and *ALB*, for quantification) were amplified. In both barcoding PCR reactions, all primers contain 5' tags used as universal linkers, allowing amplification of all loci with a single primer set in later steps. The barcoded amplified products and quantification reaction products were then pooled and purified with Zymo DNA Clean and Concentrator columns (Zymo Research, Irvine, CA).

Purified PCR products were enriched for mutations using the Boreal Genomics OnTarget platform, which is based on the previously described sequence-specific SCODA technology [11]. Polyacrylamide gel, containing short probes complementary to all 46 mutations of interest and the two wild-type control sequences, was cast into cartridges designed for the OnTarget platform. Samples were loaded into the cartridges, and synchronous, time-varying electric fields were applied to the cartridge at a fixed baseline temperature. The enrichment process focused all mutants and control sequences into an extraction well, while rejecting wild-type and off-target sequences to a waste well. Enriched DNA recovered from this process was further purified with Zymo Oligo Clean and Concentrator columns (Zymo Research, Irvine, CA).

DNA sequencing libraries were then constructed. Products were amplified and tagged with MiSeq adaptors by 35 cycles of PCR using Platinum DNA polymerase (Life Technologies, Carlsbad, CA) and universal primers, which contain 5' MiSeq adaptor tags. The PCR output from this step was then purified using the Agencourt AMPure XP kit (Beckman Coulter, Indianapolis, IN).

Samples were then quantified by qPCR using the KAPA Library Quant kit (Kapa Biosystems, Wilmington, MA), and normalized to a concentration of 2 nM. The normalized samples were then pooled in sets of six and paired-end 131 bp reads were sequenced on the MiSeq platform (Illumina, San Diego, CA).

Data Analysis

Sequencing data was analyzed in a fully automated fashion using custom analysis scripts written using BWA (Burrows-Wheeler Aligner) [12] for alignment to a custom reference library based on sequences from within the 46-mutation panel and SAM Tools (Sequence Alignment/Map) [13] for further data manipulation following alignment. Mutation quantification, quality control, and visualization were performed using scripts written in Perl, Python, and MySql and with tools including Graphviz.

Raw FastQ files from the MiSeq were first de-multiplexed by sample barcode, trimmed to retain only the endogenous regions of each molecule lying between the barcoding PCR primers, and then filtered according to the following criteria:

a) Forward and reverse reads must align to the same reference sequence

b) Both reads carry the same mutation

c) The mutation identified must be contained within the 46-mutation panel

Reads satisfying the above conditions were binned according to sample barcode and mutation. The remaining reads were then re-analyzed to determine whether they aligned to a separate reference library for the internal positive control molecules as follows:

1. The first 15 bases of the endogenous section of each read were aligned to a reduced set of reference sequences for the loci within the SCODA 46-mutation panel

2. RID barcodes were found by searching for flanking sequence specific to its locus

3. RID sequences were then removed from the endogenous sequence; the remaining endogenous sequence was then passed through the tests (a)-(c) above.

IPC reads passing the filter were corrected for sequencing errors within the RIDs and binned according to sample barcode, mutation, and unique RID sequence. The average single molecule yield through the entire workflow for each sample barcode / mutation combination was then calculated as the average number of reads over all RIDs for that barcode and mutation.

The number of input mutant molecules for each mutation within each sample was then calculated by dividing the number of mutant reads for a given barcode by the average single molecule yield for that mutation and barcode. A similar process was followed for the wild-type *COG5* and *ALB* sequences, and used to measure the total number of genomes that entered the workflow, taking into account that only 1% of these loci was amplified in the barcoding PCR reaction. Mutation abundances were calculated as the ratio of input mutant copies to total input genome copies.

Mutations were called as positive only if they passed the following criteria:

- The detected number of input mutant copies must be ≥ 1.
- The detected input mutation abundance must be greater than the average number of copies detected in historical wild-type samples plus 3 standard deviations (99.9% confidence interval, 1 tailed).
- The detected input mutation abundance must be greater than the greatest number of copies seen in the wild-type samples run in parallel with test samples.
- The detected mutation abundance must be ≥ 5% the abundance of any other mutations having an edit distance of 1 (i.e. a single base change, insertion or deletion). This criterion prevents false positive calls due to cross-talk, i.e. conversion of one mutant into another mutant due to PCR errors following multiplexed SCODA mutation enrichment. In cases where one mutation is

present at high abundance, this criterion can have a significant impact on the limit of detection for closely related mutations.

Note that the reported limit of detection is the maximum mutation abundance (%) associated with the above criteria.

Assay Characterization

Prior to running clinical samples from cancer patients, the assay was characterized for specificity, sensitivity, and reproducibility.

PCR errors in the low amplification barcoding PCR reaction can cause a low level of background mutant signal on wild-type DNA for some mutations in the panel. In order to minimize false positive calls, we applied a conservative definition of the limit of detection for each mutation, based on extensive characterization of the observed background mutant signal in experiments with reference wild-type DNA (Roche Diagnostics, Indianapolis, IN). This reference wild-type genomic DNA represents a mixture of DNA extracted from the blood of many healthy donors. In addition, a number of different lots of this reference wild-type genomic DNA, created from DNA from different sets of individuals, were tested. In total, 55 experiments were conducted on these samples, and the background mutation abundance was measured for each of the 46 mutations in the panel for each sample. The specificity limit for the assay was defined as the mean background mutant DNA signal plus 3 standard deviations from these measurements.

To characterize the sensitivity and reproducibility of the assay, experiments were performed using a model system consisting of multiple lots of reference wild-type (WT) genomic DNA (Roche Diagnostics, Indianapolis, IN), supplemented with a range of input synthetic mutant DNA sequences. Synthetic DNA sequences for each of the 46 mutations, extending past the end of the PCR priming sites, were purchased from Integrated DNA Technologies (Coralville, IA). Each of the synthetic mutant DNAs was assayed for

concentration by quantitative PCR, and diluted to a nominal concentration of 10^5 cp/µL. Equal volumes of the diluted solution for each mutation in the panel were mixed to form a solution of nominally equal concentration/copy number for all mutations. This stock was then diluted further to 100 cp/µL, and stored until further use. On the day of each experiment, a set of samples containing 0 (WT), 10, 50, or 100 nominal input copies of each mutant, each in 300 ng wild-type genomic DNA was created by serial dilution of the mixed synthetic mutant DNA stock into the reference wild-type DNA. A minimum of 10 input copies of mutant DNA was chosen for these experiments to account for sampling noise, i.e. fluctuations of the actual mutant copy number about the average, and for potential inaccuracy in the nominal mutant DNA concentration in the original stocks. The titrated samples were then analyzed with the 46-mutation SCODA assay, across multiple runs (5-12 runs per input DNA level), over multiple days and by multiple operators.

Results of the experiments are shown in Supplementary Figure 1. Mutations were readily and reproducibly quantitated above the limit of detection for all samples at 50 and 100 nominal input copies. Similarly, at 10 nominal input copies, the assay detected the input mutant DNA sequences in the vast majority of cases: in 4 out of 552 mutations tests in total (12 runs at 46 mutations per run), <1 copy of mutant DNA was observed. These dropouts may potentially be attributable to sampling noise and errors in mutant titration.

Some mutations with higher background, and higher limits of detection (e.g. *EGFR* T790M) cannot be reliably detected at 10 copies nominal input, due to the background signal observed for these mutations in wild-type DNA (see Supplementary Figure 1). This issue is less pronounced in samples with a lower overall mass of input DNA, where the background would have a lower absolute copy number (data not shown). Nearly all of the mutation calls for WT samples showed <1 input copy (478 / 552 total mutation

tests), with only 1 false positive observed over 12 runs (*KRAS* G12C, 3 copies, 0.003%; LOD 1 copy, 0.001%).

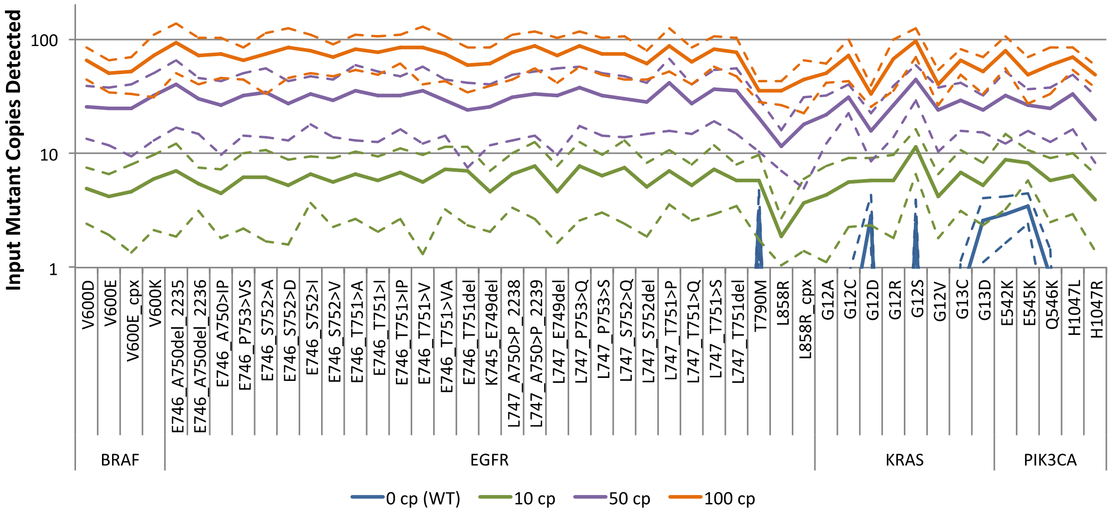

Supplementary Figure 1: Analytical characterization of the multiplexed SCODA mutation enrichment and detection assay. Synthetic mutant DNA over a range of input copies (cp) were titrated into WT genomic DNA and analyzed by the assay. Data shown are averaged over multiple runs, and show the mean (solid lines) and mean ± 1 standard deviations (dashed lines). The number of runs for each nominal input copy number was as follows: 0 cp (blue, 12 runs); 10 cp (green, 12 runs); 50 cp (purple, 6 runs) 100 cp (orange, 5 runs).

The assay was further characterized by assessment of plasma DNA from a separate set of 47 healthy donors with no clinically apparent evidence of cancer. DNA was extracted from 10 mL of plasma from each subject, and the resulting DNA was analyzed by the 46-mutation SCODA assay. As expected, no mutant DNA sequences were detected in plasma DNA for the vast majority of these subjects (43 of 47, 91%). In 4 of the 47 subjects, mutant DNA sequences were detected at very low levels (1-3 copies) approaching the limit of detection. Three of the four subjects had sufficient plasma to allow for re-testing. For two of these subjects, re-testing revealed no mutant DNA sequences. For the third subject, the same mutant DNA sequence (*KRAS* G12R) was again detected. In the absence of an orthogonal method with a similar level of analytical performance, it was not possible to characterize these cases as false positives or low-level signals corresponding to somatic mutation events that were not clinically evident. Even in the

cases where re-testing did not confirm the presence of mutant DNA sequences, the low level of mutant DNA could have been missed due to sampling noise in the second plasma sample.

1. Jones, R.P.; Stattner, S.; Sutton, P.; Dunne, D.F.; McWhirter, D.; Fenwick, S.W.; Malik, H.Z.; Poston, G.J.; Controversies in the oncosurgical management of liver limited stage IV colorectal cancer, *Surg Oncol*. **2014,** 23, 53-60.
2. Rizzo, S.; Bronte, G.; Fanale, D.; Corsini, L.; Silvestris, N.; Santini, D.; Gulotta, G.; Bazan, V.; Gebbia, N.; Fulfaro, F.; Russo, A.; Prognostic vs predictive molecular biomarkers in colorectal cancer: is KRAS and BRAF wild type status required for anti-EGFR therapy?, *Cancer Treat Rev*. **2010,** 36 Suppl 3, S56-61.
3. Liao, X.; Lochhead, P.; Nishihara, R.; Morikawa, T.; Kuchiba, A.; Yamauchi, M.; Imamura, Y.; Qian, Z.R.; Baba, Y.; Shima, K.; Sun, R.; Nosho, K.; Meyerhardt, J.A.; Giovannucci, E.; Fuchs, C.S.; Chan, A.T.; Ogino, S.; Aspirin Use, Tumor PIK3CA Mutation, and Colorectal-Cancer Survival, *New England Journal of Medicine*. **2012,** 367, 1596-1606.
4. Ogino, S.; Meyerhardt, J.A.; Irahara, N.; Niedzwiecki, D.; Hollis, D.; Saltz, L.B.; Mayer, R.J.; Schaefer, P.; Whittom, R.; Hantel, A.; Benson, A.B., 3rd; Goldberg, R.M.; Bertagnolli, M.M.; Fuchs, C.S.; KRAS mutation in stage III colon cancer and clinical outcome following intergroup trial CALGB 89803, *Clin Cancer Res*. **2009,** 15, 7322-7329.
5. De Roock, W.; Claes, B.; Bernasconi, D.; De Schutter, J.; Biesmans, B.; Fountzilas, G.; Kalogeras, K.T.; Kotoula, V.; Papamichael, D.; Laurent-Puig, P.; Penault-Llorca, F.; Rougier, P.; Vincenzi, B.; Santini, D.; Tonini, G.; Cappuzzo, F.; Frattini, M.; Molinari, F.; Saletti, P.; De Dosso, S.; Martini, M.; Bardelli, A.; Siena, S.; Sartore-Bianchi, A.; Tabernero, J.; Macarulla, T.; Di Fiore, F.; Gangloff, A.O.; Ciardiello, F.; Pfeiffer, P.; Qvortrup, C.; Hansen, T.P.; Van Cutsem, E.; Piessevaux, H.; Lambrechts, D.; Delorenzi, M.; Tejpar, S.; Effects of KRAS, BRAF, NRAS, and PIK3CA mutations on the efficacy of cetuximab plus chemotherapy in chemotherapy-refractory metastatic colorectal cancer: a retrospective consortium analysis, *Lancet Oncol*. **2010,** 11, 753-762.
6. Comprehensive molecular characterization of human colon and rectal cancer, *Nature*. **2012,** 487, 330-337.
7. Comprehensive molecular profiling of lung adenocarcinoma, *Nature*. **2014,** 511, 543-550.
8. Sorensen, B.S.; Wu, L.; Wei, W.; Tsai, J.; Weber, B.; Nexo, E.; Meldgaard, P.; Monitoring of epidermal growth factor receptor tyrosine kinase inhibitor-sensitizing and resistance mutations in the plasma DNA of patients with advanced non-small cell lung cancer during treatment with erlotinib, *Cancer*. **2014,**
9. Lynch, T.J.; Bell, D.W.; Sordella, R.; Gurubhagavatula, S.; Okimoto, R.A.; Brannigan, B.W.; Harris, P.L.; Haserlat, S.M.; Supko, J.G.; Haluska, F.G.; Louis, D.N.; Christiani, D.C.; Settleman, J.; Haber, D.A.; Activating mutations in the epidermal growth factor receptor underlying responsiveness of non-small-cell lung cancer to gefitinib, *N Engl J Med*. **2004,** 350, 2129-2139.

10. Sun, Y.; Ren, Y.; Fang, Z.; Li, C.; Fang, R.; Gao, B.; Han, X.; Tian, W.; Pao, W.; Chen, H.; Ji, H.; Lung adenocarcinoma from East Asian never-smokers is a disease largely defined by targetable oncogenic mutant kinases, *J Clin Oncol*. **2010,** 28, 4616-4620.
11. Thompson, J.D.; Shibahara, G.; Rajan, S.; Pel, J.; Marziali, A.; Winnowing DNA for rare sequences: highly specific sequence and methylation based enrichment, *PLoS One*. **2012,** 7, e31597.
12. Li, H.; Durbin, R.; Fast and accurate short read alignment with Burrows-Wheeler transform, *Bioinformatics*. **2009,** 25, 1754-1760.
13. Li, H.; Handsaker, B.; Wysoker, A.; Fennell, T.; Ruan, J.; Homer, N.; Marth, G.; Abecasis, G.; Durbin, R.; The Sequence Alignment/Map format and SAMtools, *Bioinformatics*. **2009,** 25, 2078-2079.
